# Redetermination of tris­(*N*,*N*-diethyl­dithio­carbamato)anti­mony(III)

**DOI:** 10.1107/S1600536809005303

**Published:** 2009-02-21

**Authors:** Mei Que, Yu Chuan Zhang, Zhao Di Liu, Hong Ping Zhou

**Affiliations:** a Department of Chemistry Anhui University, Hefei 230039, People’s Republic of China, and Key Laboratory of Enviromentally-Friendly Polymer Materials of Anhui Province, Hefei 230039, People’s Republic of China

## Abstract

The title compound, [Sb(C_5_H_10_NS_2_)_3_], was synthesized from Sb_2_O_3_, diethyl­amine, carbon dis­ulfide, hydro­chloric acid and sodium hydroxide. The structure has been published previously but H atoms were not included in the model [Raston & White (1976 ▶). *Chem. Soc. Dalton Trans*. p. 791]. The current determination has significantly higher precision than the original work. The complex has three ligands. The Sb atom is coordinated by three bidentate diethyl­dithio­carbamate groups, two in an almost planar fashion and the third perpendicular to that plane with a dihedral angle of 86.429 (13)°. One ethyl group is disordered over two positions of equal occupancy.

## Related literature

For applications of dithio­carbamates, see: Fujii & Yoshimura (2000 ▶); Stary *et al.* (1992 ▶); Pazukhina *et al.* (1997 ▶). For the extraction efficiency of dithio­carbamate complexes in the presence of neutral N, S, O and P donor mol­ecules, see: Ooi & Fernando (1967 ▶). For nitro­gen donor adducts of dithio­carbamate complexes, see: O’Brien *et al.* (1992 ▶, 1998 ▶); Chunggaze *et al.* (1997 ▶); Bessergenev *et al.* (1996 ▶, 1997 ▶); Hovel (1975 ▶). For complexes with post-transition metals, see: Coucouvanis (1979 ▶) and for complexes involving Te(IV), Te(II) and Se(II) centres, see: Husebye & Svaeren (1973 ▶); Rout *et al.* (1983 ▶).
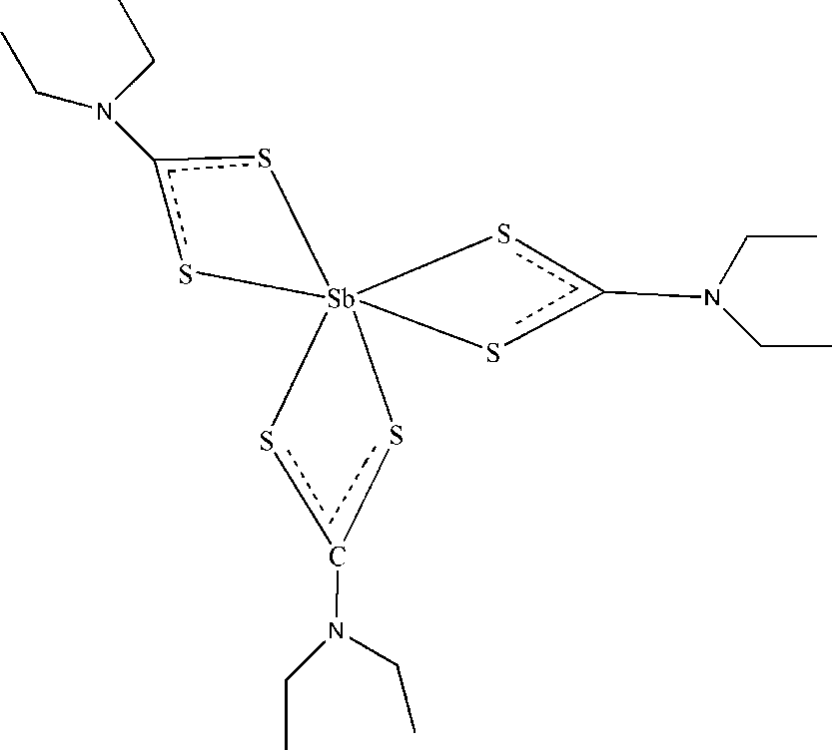


## Experimental

### 

#### Crystal data

[Sb(C_5_H_10_NS_2_)_3_]
*M* *_r_* = 566.53
Monoclinic, 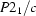

*a* = 12.6454 (2) Å
*b* = 13.6217 (2) Å
*c* = 14.6731 (2) Å
β = 99.858 (1)°
*V* = 2490.15 (6) Å^3^
*Z* = 4
Mo *K*α radiation
μ = 1.62 mm^−1^
*T* = 296 K
0.26 × 0.21 × 0.21 mm

#### Data collection

Bruker SMART CCD area-detector diffractometer
Absorption correction: multi-scan (*SADABS*; Bruker, 2001 ▶) *T*_min_ = 0.679, *T*_max_ = 0.817 (expected range = 0.591–0.712)
24761 measured reflections
5746 independent reflections
4947 reflections with *I* > 2σ(*I*)
*R*_int_ = 0.021

#### Refinement

*R*[*F* ^2^ > 2σ(*F* ^2^)] = 0.021
*wR*(*F* ^2^) = 0.059
*S* = 1.00
5746 reflections
253 parameters
H-atom parameters constrained
Δρ_max_ = 0.38 e Å^−3^
Δρ_min_ = −0.29 e Å^−3^

### 

Data collection: *SMART* (Bruker, 2007 ▶); cell refinement: *SAINT* (Bruker, 2007 ▶); data reduction: *SAINT* program(s) used to solve structure: *SHELXS97* (Sheldrick, 2008 ▶); program(s) used to refine structure: *SHELXL97* (Sheldrick, 2008 ▶); molecular graphics: *SHELXTL* (Sheldrick, 2008 ▶); software used to prepare material for publication: *SHELXTL*.

## Supplementary Material

Crystal structure: contains datablocks global, I. DOI: 10.1107/S1600536809005303/fi2068sup1.cif

Structure factors: contains datablocks I. DOI: 10.1107/S1600536809005303/fi2068Isup2.hkl

Additional supplementary materials:  crystallographic information; 3D view; checkCIF report

## Figures and Tables

**Table 1:** Selected bond lengths (Å)

Sb1—S5	2.4842 (5)
Sb1—S3	2.6238 (5)
Sb1—S2	2.6328 (5)
Sb1—S1	2.8805 (6)
Sb1—S4	2.8938 (5)

**Fig. 1.**
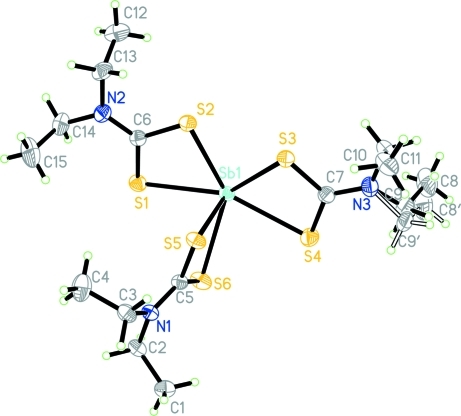
The structure of (I), showing 30% probability displacement ellipsoids and the atom-numbering scheme.

## supplementary crystallographic information

### Comment

Dithiocarbamates have found wide practical application as antioxidants and lubricants, as vulcanizing and NO-trapping agents (Fujii *et al.*, 2000), as agents for the froth flotation process of sulfide minerals and for the liquid-liquid extraction of transition metals (Stary *et al.*, 1992; Pazukhina *et al.*, 1997). It has also been found that the extraction efficiency of dithiocarbamate complexes rises in the presence of neutral N, S, O, P-donor molecules, which could potentially imply formation of adducts (Ooi *et al.*, 1967). Besides that, nitrogen donor adducts of dithiocarbamate complexes are also widely used in the preparation of thin semiconductor (O'Brien *et al.*, 1992; Chunggaze *et al.*, 1997; O'Brien *et al.*, 1998) and electroluminescent (Bessergenev *et al.*, 1996; Bessergenev *et al.*, 1997) films of transition metal sulfides, the basis of electronics and solar cell technology (Hovel, 1975). The dithiocarbamate anion (*R*_1_*R*_2_NCS^2-^=I^-^) is known to be a strong nucleophile and to form stable complexs with many post-transition metals (Coucouvanis, 1979). Thus, complexes involving Te(IV), Te(II) and Se(II) centres have been reported, and structural studies on these have shown the presence of bidentate chelating ligands (Husebye *et al.*, 1973; Rout *et al.*, 1983). We have synthesized the title compound, C_15_H_30_N_3_S_6_Sb, and report here its crystal structure. The structure had been reported earlier (Raston & White, 1976), The syntheses and application of the crystal haven't been described in that paper. They mentioned simply that they examined samples of the antimony derivatives recrystallized from benzene solution. The crystal is monoclinic, Z=4, a=14.665 (5) Å, b=13.619 (5)Å, c=12.642 (4)Å, β=99.86 (4)°. These data of the crystal is similar to our crystal.but no hydrogen atoms were included in their structure model. The molecular structure and the atom-numbering scheme of the title compound are shown in Fig. 1. In the molecule, all bond lengths and angles agree well with values found in literature Table 1. The Sb atom is coordinated by three bidentate diethyldithiocarbamato groups, two groups in an almost planar fashion, the thirs group is perpendicular to that plane with a dihedral angle of 86.429 (13)°.

### Experimental

Water (200 ml), sodium hydroxide (4 g, 0.1 mol) and diethylamine (7.3 g, 0.1 mol) were added to a three-neck flask in an icewater bath under stirring. Carbon disulfide (7.8 g, 0.1 mol) was added dropwise into this solution during twenty minutes and the mixture was allowed to react for four hours yielding a light yellow liquid. Antimony trioxide (4.6 g, 0.016 mol) was dissolved in hydrochloric acid. The solution was added dropwise into the diethyl dithiocarbamate natrium under stirring and it was confirmed that the resulting solution was acidic. From this solution, a yellow deposit was obtained. It was collected by vacuum filtration, washed with a large amount of water and dried in air. Yellow single crystals were obtained after two weeks upon evaporation of a solution of the reaction product in a mixture of chloroform (5 ml) and methanol (30 ml).

### Refinement

Atom C8 and C9 were found to be disordered over two positions with the same site-occupancy factors (0.50/0.50). All hydrogen atoms were placed in geometrically idealized positions and constrained to ride on their parent atoms, with C—H = 0.97, 0.96(–CH_3_) Å and *U*_iso_(H) = 1.2*U*_eq_(C), 1.5 *U*_eq_(–CH_3_), respectively.

### Crystal data

**Table:**

[Sb(C_5_H_10_NS_2_)_3_]	*F*(000) = 1152
*M**_r_* = 566.53	*D*_x_ = 1.511 Mg m^−^^3^
Monoclinic, *P*2_1_/*c*	Mo *K*α radiation, λ = 0.71073 Å
Hall symbol: -P 2ybc	Cell parameters from 9907 reflections
*a* = 12.6454 (2) Å	θ = 2.2–27.5°
*b* = 13.6217 (2) Å	µ = 1.62 mm^−^^1^
*c* = 14.6731 (2) Å	*T* = 296 K
β = 99.858 (1)°	Block, yellow
*V* = 2490.15 (6) Å^3^	0.26 × 0.21 × 0.21 mm
*Z* = 4	

### Data collection

**Table:**

Bruker SMART CCD area-detector diffractometer	5746 independent reflections
Radiation source: fine-focus sealed tube	4947 reflections with *I* > 2σ(*I*)
graphite	*R*_int_ = 0.021
φ and ω scans	θ_max_ = 27.6°, θ_min_ = 1.6°
Absorption correction: multi-scan (*SADABS*; Bruker, 2001)	*h* = −16→16
*T*_min_ = 0.679, *T*_max_ = 0.817	*k* = −17→17
24761 measured reflections	*l* = −18→19

### Refinement

**Table:**

Refinement on *F*^2^	Secondary atom site location: difference Fourier map
Least-squares matrix: full	Hydrogen site location: inferred from neighbouring sites
*R*[*F*^2^ > 2σ(*F*^2^)] = 0.021	H-atom parameters constrained
*wR*(*F*^2^) = 0.059	*w* = 1/[σ^2^(*F*_o_^2^) + (0.0366*P*)^2^ + 0.1807*P*] where *P* = (*F*_o_^2^ + 2*F*_c_^2^)/3
*S* = 1.00	(Δ/σ)_max_ = 0.003
5746 reflections	Δρ_max_ = 0.38 e Å^−^^3^
253 parameters	Δρ_min_ = −0.29 e Å^−^^3^
0 restraints	Extinction correction: *SHELXL97* (Sheldrick, 2008), Fc^*^=kFc[1+0.001xFc^2^λ^3^/sin(2θ)]^-1/4^
Primary atom site location: structure-invariant direct methods	Extinction coefficient: 0.0072 (2)

### Special details

**Table:**

Geometry. All esds (except the esd in the dihedral angle between two l.s. planes) are estimated using the full covariance matrix. The cell esds are taken into account individually in the estimation of esds in distances, angles and torsion angles; correlations between esds in cell parameters are only used when they are defined by crystal symmetry. An approximate (isotropic) treatment of cell esds is used for estimating esds involving l.s. planes.
Refinement. Refinement of F^2^ against ALL reflections. The weighted R-factor wR and goodness of fit S are based on F^2^, conventional R-factors R are based on F, with F set to zero for negative F^2^. The threshold expression of F^2^ > 2sigma(F^2^) is used only for calculating R-factors(gt) etc. and is not relevant to the choice of reflections for refinement. R-factors based on F^2^ are statistically about twice as large as those based on F, and R- factors based on ALL data will be even larger.

### Fractional atomic coordinates and isotropic or equivalent isotropic displacement parameters (Å^2^)

**Table:**

	*x*	*y*	*z*	*U*_iso_*/*U*_eq_	Occ. (<1)
Sb1	0.664203 (10)	0.489969 (8)	0.544433 (8)	0.03895 (6)	
S5	0.82733 (4)	0.47481 (3)	0.47217 (3)	0.04547 (12)	
S2	0.59116 (4)	0.32605 (3)	0.46160 (3)	0.04974 (12)	
S1	0.55799 (5)	0.51072 (3)	0.35546 (4)	0.04951 (13)	
S3	0.73733 (4)	0.34808 (3)	0.65900 (3)	0.04951 (12)	
S6	0.75061 (4)	0.67918 (4)	0.48886 (4)	0.05794 (14)	
S4	0.78957 (5)	0.55135 (4)	0.71886 (3)	0.05585 (13)	
N1	0.88457 (11)	0.62440 (10)	0.37638 (9)	0.0413 (3)	
N3	0.81757 (14)	0.39580 (12)	0.83190 (10)	0.0558 (4)	
N2	0.50310 (13)	0.33416 (11)	0.28478 (10)	0.0495 (4)	
C2	0.89233 (16)	0.72713 (14)	0.34826 (13)	0.0503 (4)	
H2A	0.8274	0.7617	0.3560	0.060*	
H2B	0.8984	0.7297	0.2833	0.060*	
C5	0.82576 (13)	0.59892 (13)	0.43964 (11)	0.0397 (4)	
C6	0.54573 (13)	0.38630 (13)	0.35802 (12)	0.0413 (4)	
C7	0.78502 (15)	0.43078 (14)	0.74662 (12)	0.0457 (4)	
C3	0.94447 (15)	0.55426 (14)	0.32952 (12)	0.0495 (4)	
H3A	0.9652	0.4986	0.3699	0.059*	
H3B	1.0094	0.5852	0.3168	0.059*	
C10	0.82177 (18)	0.29025 (16)	0.85339 (14)	0.0616 (6)	
H10A	0.7619	0.2575	0.8152	0.074*	
H10B	0.8146	0.2810	0.9176	0.074*	
C13	0.49020 (17)	0.22693 (15)	0.28767 (15)	0.0596 (5)	
H13A	0.5502	0.1991	0.3298	0.071*	
H13B	0.4913	0.2001	0.2266	0.071*	
C12	0.38672 (19)	0.19757 (17)	0.31864 (19)	0.0797 (7)	
H12A	0.3880	0.2189	0.3812	0.120*	
H12B	0.3791	0.1275	0.3154	0.120*	
H12C	0.3273	0.2277	0.2790	0.120*	
C14	0.46194 (18)	0.38151 (18)	0.19505 (13)	0.0669 (6)	
H14A	0.4376	0.4473	0.2060	0.080*	
H14B	0.4008	0.3446	0.1635	0.080*	
C1	0.98809 (18)	0.77734 (16)	0.40466 (15)	0.0672 (6)	
H1A	0.9786	0.7809	0.4681	0.101*	
H1B	0.9947	0.8425	0.3812	0.101*	
H1C	1.0519	0.7406	0.4005	0.101*	
C4	0.8789 (3)	0.51864 (19)	0.24026 (17)	0.0816 (8)	
H4A	0.8182	0.4822	0.2533	0.122*	
H4B	0.9223	0.4771	0.2088	0.122*	
H4C	0.8544	0.5739	0.2018	0.122*	
C11	0.9249 (2)	0.24437 (17)	0.83695 (16)	0.0733 (7)	
H11A	0.9262	0.2427	0.7717	0.110*	
H11B	0.9300	0.1787	0.8610	0.110*	
H11C	0.9845	0.2824	0.8676	0.110*	
C15	0.5461 (2)	0.3870 (2)	0.13415 (16)	0.0965 (9)	
H15A	0.6062	0.4244	0.1647	0.145*	
H15B	0.5165	0.4182	0.0768	0.145*	
H15C	0.5693	0.3219	0.1221	0.145*	
C9	0.8390 (10)	0.4544 (13)	0.9120 (12)	0.072 (3)	0.50
H9A	0.8534	0.5205	0.8927	0.087*	0.50
H9B	0.9048	0.4303	0.9492	0.087*	0.50
C8	0.7567 (14)	0.4614 (9)	0.9733 (10)	0.104 (4)	0.50
H8A	0.6917	0.4886	0.9391	0.156*	0.50
H8B	0.7828	0.5031	1.0250	0.156*	0.50
H8C	0.7425	0.3971	0.9952	0.156*	0.50
C9'	0.8734 (10)	0.4662 (11)	0.9077 (10)	0.060 (2)	0.50
H9'1	0.9315	0.4325	0.9473	0.072*	0.50
H9'2	0.9031	0.5220	0.8797	0.072*	0.50
C8'	0.7900 (13)	0.5005 (9)	0.9641 (9)	0.101 (4)	0.50
H8'1	0.7337	0.5349	0.9246	0.151*	0.50
H8'2	0.8232	0.5436	1.0124	0.151*	0.50
H8'3	0.7605	0.4447	0.9908	0.151*	0.50

### Atomic displacement parameters (Å^2^)

**Table:**

	*U*^11^	*U*^22^	*U*^33^	*U*^12^	*U*^13^	*U*^23^
Sb1	0.04014 (8)	0.03772 (8)	0.04160 (8)	−0.00181 (4)	0.01435 (6)	−0.00116 (4)
S5	0.0466 (3)	0.0374 (2)	0.0571 (3)	0.00373 (19)	0.0225 (2)	0.00350 (19)
S2	0.0617 (3)	0.0378 (2)	0.0487 (3)	−0.0062 (2)	0.0063 (2)	0.00249 (18)
S1	0.0591 (3)	0.0388 (2)	0.0515 (3)	0.0014 (2)	0.0121 (2)	0.00393 (18)
S3	0.0582 (3)	0.0423 (2)	0.0471 (3)	−0.0066 (2)	0.0065 (2)	0.00099 (19)
S6	0.0706 (3)	0.0388 (3)	0.0737 (3)	0.0029 (2)	0.0387 (3)	−0.0037 (2)
S4	0.0677 (3)	0.0433 (3)	0.0550 (3)	−0.0018 (2)	0.0059 (2)	−0.0053 (2)
N1	0.0389 (8)	0.0427 (8)	0.0442 (8)	−0.0013 (6)	0.0123 (6)	0.0028 (6)
N3	0.0663 (11)	0.0571 (10)	0.0432 (9)	−0.0020 (8)	0.0072 (8)	0.0013 (7)
N2	0.0515 (9)	0.0483 (9)	0.0484 (9)	0.0001 (7)	0.0078 (7)	−0.0079 (7)
C2	0.0534 (11)	0.0477 (10)	0.0521 (10)	−0.0010 (9)	0.0161 (9)	0.0103 (8)
C5	0.0378 (9)	0.0376 (9)	0.0450 (9)	−0.0025 (7)	0.0110 (7)	−0.0014 (7)
C6	0.0378 (9)	0.0430 (9)	0.0447 (9)	0.0016 (7)	0.0117 (8)	−0.0021 (7)
C7	0.0430 (10)	0.0503 (11)	0.0451 (10)	−0.0012 (8)	0.0116 (8)	−0.0015 (8)
C3	0.0456 (10)	0.0537 (11)	0.0538 (10)	0.0049 (9)	0.0219 (9)	0.0023 (9)
C10	0.0716 (14)	0.0657 (13)	0.0491 (11)	−0.0094 (11)	0.0145 (10)	0.0162 (10)
C13	0.0644 (14)	0.0482 (11)	0.0667 (12)	−0.0050 (10)	0.0128 (10)	−0.0186 (10)
C12	0.0692 (15)	0.0656 (15)	0.1058 (19)	−0.0162 (13)	0.0191 (14)	−0.0159 (14)
C14	0.0747 (15)	0.0742 (15)	0.0472 (11)	0.0074 (12)	−0.0027 (10)	−0.0055 (10)
C1	0.0729 (15)	0.0589 (13)	0.0704 (14)	−0.0189 (11)	0.0138 (12)	0.0004 (10)
C4	0.098 (2)	0.0876 (18)	0.0591 (15)	0.0177 (15)	0.0135 (14)	−0.0195 (12)
C11	0.0946 (19)	0.0606 (14)	0.0697 (14)	0.0029 (13)	0.0287 (13)	0.0149 (11)
C15	0.132 (2)	0.105 (2)	0.0598 (14)	0.0332 (19)	0.0349 (16)	0.0051 (13)
C9	0.073 (8)	0.074 (6)	0.062 (5)	−0.013 (5)	−0.010 (5)	0.003 (4)
C8	0.158 (11)	0.104 (8)	0.057 (4)	0.014 (7)	0.037 (5)	−0.013 (5)
C9'	0.072 (7)	0.060 (4)	0.042 (3)	−0.011 (4)	−0.005 (4)	−0.008 (3)
C8'	0.138 (11)	0.114 (10)	0.052 (4)	0.027 (7)	0.020 (5)	−0.007 (5)

### Geometric parameters (Å, °)

**Table:**

Sb1—S5	2.4842 (5)	C13—H13B	0.9700
Sb1—S3	2.6238 (5)	C12—H12A	0.9600
Sb1—S2	2.6328 (5)	C12—H12B	0.9600
Sb1—S1	2.8805 (6)	C12—H12C	0.9600
Sb1—S4	2.8938 (5)	C14—C15	1.504 (3)
S5—C5	1.7559 (17)	C14—H14A	0.9700
S2—C6	1.7362 (18)	C14—H14B	0.9700
S1—C6	1.7028 (18)	C1—H1A	0.9600
S3—C7	1.7378 (19)	C1—H1B	0.9600
S6—C5	1.6896 (17)	C1—H1C	0.9600
S4—C7	1.696 (2)	C4—H4A	0.9600
N1—C5	1.331 (2)	C4—H4B	0.9600
N1—C3	1.462 (2)	C4—H4C	0.9600
N1—C2	1.467 (2)	C11—H11A	0.9600
N3—C7	1.336 (2)	C11—H11B	0.9600
N3—C9	1.408 (17)	C11—H11C	0.9600
N3—C10	1.471 (3)	C15—H15A	0.9600
N3—C9'	1.545 (13)	C15—H15B	0.9600
N2—C6	1.324 (2)	C15—H15C	0.9600
N2—C13	1.471 (2)	C9—C8	1.49 (2)
N2—C14	1.478 (2)	C9—H9A	0.9700
C2—C1	1.509 (3)	C9—H9B	0.9700
C2—H2A	0.9700	C8—H8A	0.9600
C2—H2B	0.9700	C8—H8B	0.9600
C3—C4	1.505 (3)	C8—H8C	0.9600
C3—H3A	0.9700	C9'—C8'	1.52 (2)
C3—H3B	0.9700	C9'—H9'1	0.9700
C10—C11	1.503 (3)	C9'—H9'2	0.9700
C10—H10A	0.9700	C8'—H8'1	0.9600
C10—H10B	0.9700	C8'—H8'2	0.9600
C13—C12	1.511 (3)	C8'—H8'3	0.9600
C13—H13A	0.9700		
			
S5—Sb1—S3	89.136 (16)	N2—C13—H13B	109.2
S5—Sb1—S2	89.071 (16)	C12—C13—H13B	109.2
S3—Sb1—S2	74.239 (14)	H13A—C13—H13B	107.9
S5—Sb1—S1	83.205 (17)	C13—C12—H12A	109.5
S3—Sb1—S1	138.085 (14)	C13—C12—H12B	109.5
S2—Sb1—S1	64.525 (14)	H12A—C12—H12B	109.5
S5—Sb1—S4	91.853 (17)	C13—C12—H12C	109.5
S3—Sb1—S4	64.313 (15)	H12A—C12—H12C	109.5
S2—Sb1—S4	138.518 (15)	H12B—C12—H12C	109.5
S1—Sb1—S4	156.580 (15)	N2—C14—C15	111.95 (18)
C5—S5—Sb1	93.69 (5)	N2—C14—H14A	109.2
C6—S2—Sb1	92.32 (6)	C15—C14—H14A	109.2
C6—S1—Sb1	84.85 (6)	N2—C14—H14B	109.2
C7—S3—Sb1	92.07 (7)	C15—C14—H14B	109.2
C7—S4—Sb1	84.06 (6)	H14A—C14—H14B	107.9
C5—N1—C3	123.63 (15)	C2—C1—H1A	109.5
C5—N1—C2	121.14 (15)	C2—C1—H1B	109.5
C3—N1—C2	115.22 (14)	H1A—C1—H1B	109.5
C7—N3—C9	124.3 (8)	C2—C1—H1C	109.5
C7—N3—C10	122.90 (16)	H1A—C1—H1C	109.5
C9—N3—C10	112.4 (7)	H1B—C1—H1C	109.5
C7—N3—C9'	118.9 (6)	C3—C4—H4A	109.5
C9—N3—C9'	17.9 (8)	C3—C4—H4B	109.5
C10—N3—C9'	117.2 (6)	H4A—C4—H4B	109.5
C6—N2—C13	122.77 (16)	C3—C4—H4C	109.5
C6—N2—C14	121.42 (16)	H4A—C4—H4C	109.5
C13—N2—C14	115.78 (15)	H4B—C4—H4C	109.5
N1—C2—C1	111.36 (16)	C10—C11—H11A	109.5
N1—C2—H2A	109.4	C10—C11—H11B	109.5
C1—C2—H2A	109.4	H11A—C11—H11B	109.5
N1—C2—H2B	109.4	C10—C11—H11C	109.5
C1—C2—H2B	109.4	H11A—C11—H11C	109.5
H2A—C2—H2B	108.0	H11B—C11—H11C	109.5
N1—C5—S6	123.48 (13)	C14—C15—H15A	109.5
N1—C5—S5	117.32 (12)	C14—C15—H15B	109.5
S6—C5—S5	119.20 (9)	H15A—C15—H15B	109.5
N2—C6—S1	122.83 (14)	C14—C15—H15C	109.5
N2—C6—S2	119.00 (14)	H15A—C15—H15C	109.5
S1—C6—S2	118.17 (10)	H15B—C15—H15C	109.5
N3—C7—S4	123.55 (14)	N3—C9—C8	119.0 (9)
N3—C7—S3	118.29 (15)	N3—C9—H9A	107.6
S4—C7—S3	118.16 (10)	C8—C9—H9A	107.6
N1—C3—C4	111.55 (17)	N3—C9—H9B	107.6
N1—C3—H3A	109.3	C8—C9—H9B	107.6
C4—C3—H3A	109.3	H9A—C9—H9B	107.0
N1—C3—H3B	109.3	C8'—C9'—N3	107.9 (9)
C4—C3—H3B	109.3	C8'—C9'—H9'1	110.1
H3A—C3—H3B	108.0	N3—C9'—H9'1	110.1
N3—C10—C11	111.87 (17)	C8'—C9'—H9'2	110.1
N3—C10—H10A	109.2	N3—C9'—H9'2	110.1
C11—C10—H10A	109.2	H9'1—C9'—H9'2	108.4
N3—C10—H10B	109.2	C9'—C8'—H8'1	109.5
C11—C10—H10B	109.2	C9'—C8'—H8'2	109.5
H10A—C10—H10B	107.9	H8'1—C8'—H8'2	109.5
N2—C13—C12	112.18 (17)	C9'—C8'—H8'3	109.5
N2—C13—H13A	109.2	H8'1—C8'—H8'3	109.5
C12—C13—H13A	109.2	H8'2—C8'—H8'3	109.5
			
S3—Sb1—S5—C5	−150.76 (6)	C14—N2—C6—S2	179.31 (14)
S2—Sb1—S5—C5	134.99 (6)	Sb1—S1—C6—N2	−176.81 (15)
S1—Sb1—S5—C5	70.53 (6)	Sb1—S1—C6—S2	3.31 (9)
S4—Sb1—S5—C5	−86.50 (6)	Sb1—S2—C6—N2	176.50 (13)
S5—Sb1—S2—C6	−80.83 (6)	Sb1—S2—C6—S1	−3.61 (10)
S3—Sb1—S2—C6	−170.20 (6)	C9—N3—C7—S4	13.3 (6)
S1—Sb1—S2—C6	2.08 (6)	C10—N3—C7—S4	−174.62 (15)
S4—Sb1—S2—C6	−172.58 (6)	C9'—N3—C7—S4	−6.7 (6)
S5—Sb1—S1—C6	90.09 (6)	C9—N3—C7—S3	−167.7 (6)
S3—Sb1—S1—C6	9.03 (7)	C10—N3—C7—S3	4.3 (3)
S2—Sb1—S1—C6	−2.13 (6)	C9'—N3—C7—S3	172.3 (6)
S4—Sb1—S1—C6	168.95 (6)	Sb1—S4—C7—N3	−170.09 (16)
S5—Sb1—S3—C7	99.33 (6)	Sb1—S4—C7—S3	10.93 (9)
S2—Sb1—S3—C7	−171.40 (6)	Sb1—S3—C7—N3	168.95 (14)
S1—Sb1—S3—C7	178.14 (6)	Sb1—S3—C7—S4	−12.02 (10)
S4—Sb1—S3—C7	6.85 (6)	C5—N1—C3—C4	92.1 (2)
S5—Sb1—S4—C7	−95.21 (6)	C2—N1—C3—C4	−87.3 (2)
S3—Sb1—S4—C7	−7.06 (6)	C7—N3—C10—C11	85.0 (2)
S2—Sb1—S4—C7	−4.51 (7)	C9—N3—C10—C11	−102.1 (6)
S1—Sb1—S4—C7	−172.31 (7)	C9'—N3—C10—C11	−83.1 (6)
C5—N1—C2—C1	92.8 (2)	C6—N2—C13—C12	86.1 (2)
C3—N1—C2—C1	−87.77 (19)	C14—N2—C13—C12	−91.9 (2)
C3—N1—C5—S6	−176.11 (13)	C6—N2—C14—C15	92.1 (2)
C2—N1—C5—S6	3.2 (2)	C13—N2—C14—C15	−89.8 (2)
C3—N1—C5—S5	3.6 (2)	C7—N3—C9—C8	100.7 (13)
C2—N1—C5—S5	−177.00 (13)	C10—N3—C9—C8	−72.1 (15)
Sb1—S5—C5—N1	−163.57 (12)	C9'—N3—C9—C8	178 (5)
Sb1—S5—C5—S6	16.20 (10)	C7—N3—C9'—C8'	98.3 (9)
C13—N2—C6—S1	−178.50 (14)	C9—N3—C9'—C8'	−15 (4)
C14—N2—C6—S1	−0.6 (3)	C10—N3—C9'—C8'	−93.1 (10)
C13—N2—C6—S2	1.4 (2)		
